# RNA-Seq Based Transcriptome Analysis of Hepatitis E Virus (HEV) and Hepatitis B Virus (HBV) Replicon Transfected Huh-7 Cells

**DOI:** 10.1371/journal.pone.0087835

**Published:** 2014-02-05

**Authors:** Neetu Jagya, Satya Pavan Kumar Varma, Deepshi Thakral, Prashant Joshi, Hemlata Durgapal, Subrat Kumar Panda

**Affiliations:** Department of Pathology, All India Institute of Medical Sciences, Ansari Nagar, New Delhi, India; University of Cincinnati College of Medicine, United States of America

## Abstract

Pathogenesis of hepatitis B virus (HBV) and hepatitis E virus (HEV) infection is as varied as they appear similar; while HBV causes an acute and/or chronic liver disease and hepatocellular carcinoma, HEV mostly causes an acute self-limiting disease. In both infections, host responses are crucial in disease establishment and/or virus clearance. In the wake of worsening prognosis described during HEV super-infection over chronic HBV hepatitis, we investigated the host responses by studying alterations in gene expression in liver cells (Huh-7 cell line) by transfection with HEV replicon only (HEV-only), HBV replicon only (HBV-only) and both HBV and HEV replicons (HBV+HEV). Virus replication was validated by strand-specific real-time RT-PCR for HEV and HBsAg ELISA of the culture supernatants for HBV. Indirect immunofluorescence for the respective viral proteins confirmed infection. Transcription profiling was carried out by RNA Sequencing (RNA-Seq) analysis of the poly-A enriched RNA from the transfected cells. Averages of 600 million bases within 5.6 million reads were sequenced in each sample and ∼15,800 genes were mapped with at least one or more reads. A total of 461 genes in HBV+HEV, 408 in HBV-only and 306 in HEV-only groups were differentially expressed as compared to mock transfection control by two folds (p<0.05) or more. Majority of the significant genes with altered expression clustered into immune-associated, signal transduction, and metabolic process categories. Differential gene expression of functionally important genes in these categories was also validated by real-time RT-PCR based relative gene-expression analysis. To our knowledge, this is the first report of *in vitro* replicon transfected RNA-Seq based transcriptome analysis to understand the host responses against HEV and HBV.

## Introduction

Hepatitis E virus (HEV) is a small, 27–34 nm, non-enveloped, single stranded, positive-sense, ∼7.2 kb RNA containing virus, which enters the host mostly through oral route [1]. Hepatitis B virus (HBV) is a parenterally transmitted 40–45 nm, enveloped, partially double stranded circular 3.2 kb DNA containing virus [2]. HBV infection is widespread and involves over 350 million people worldwide [3] and is a major cause of chronic hepatitis. A large proportion of these chronically infected individuals reside in areas endemic for HEV infection.

Hepatitis E virus has emerged as a global pathogen affecting the food chain [4] and besides causing epidemics and high mortality in pregnancy [5], it has recently been associated with chronicity in solid organ recipients and immunosuppressed patients [6]. HEV has also been described to cause super-infection in chronic HBV associated liver disease patients [7], [8], [9], [10], [11], [12], [13], [14], [15] accounting for 20% of cases in regions endemic for HEV [13]. The reason for the rapid decompensation in HEV super-infection over chronic HBV infection is not understood. Both viruses are supposed to be non-cytopathic. Therefore, the alteration in initial host cell response, and subsequently acquired defense mechanisms against virus infection might be playing important roles. Detailed understanding of pathobiology of HEV super-infection is indispensable in this regard.

Evidence indicates the involvement of cell-mediated immune responses in the pathogenesis of both HEV and HBV infection [16], [17]. However, the host-pathogen interaction is initiated much before the acquired defense mechanism sets in. It is therefore important to understand the initial host responses that can modify the outcome of infection. To gain a better understanding of the immediate changes that occur after infection, in the absence of external response modifiers present *in vivo* and bypass the quantitative restrictions present in proteomic tools, we investigated the host responses induced by HEV replicon when transfected alone and when transfected in HBV infected Huh-7 cells established by HBV 1.3mer replicon transfection. We used RNA sequencing (RNA-Seq) based transcriptome analysis and real-time PCR based relative gene expression validation on cells transfected with HEV-only (HEV), HBV-only (HBV) and HBV+HEV replicons. Analysis for differential gene expression revealed several innate immune response, signal transduction, cell survival and metabolism associated genes. The significance of this altered gene expression in the likely outcome of both HEV infection and its super-infection on HBV is discussed.

## Materials and Methods

### HEV and HBV Clones

Full length capped and polyadenylated HEV genomic RNA was obtained by *in vitro* transcription from the full length HEV cDNA clone pSGHEV-HB (FJ457024) (Genotype 1) [1] using mMessage mMachine T7 ultra kit (Life Technologies, USA), as described earlier [1]. The reconstituted RNA was quantified using Nanodrop spectrophotometer (Thermo Scientific, USA) and stored at −80°C till further use. An over-length 1.3mer HBV genotype-A replicon in pcDNA3 null vector was constructed from pRLnull(77–1246)WTSPGE and pRLnullΔCMVWT1.86mer (Figure S1). pcDNA3ΔCMVHBVWT-1.3mer (HBV1.3mer) and pcDNA3-null vector DNA (used for mock transfection in the present study) were prepared using Plasmid Mini kit (Qiagen, Germany) as per manufacturer’s instructions. The reconstituted DNA was quantified using Nanodrop spectrophotometer and stored at −20°C till further use.

### Cell Culture and Replicon Transfections

Human hepatoma cell line (Huh-7) was maintained in Dulbeco’s Modified Eagle’s Medium (DMEM) (Invitrogen, USA) supplemented with 10% heat inactivated fetal bovine serum (Invitrogen, USA), and 1X Antibiotic-Antimycotic (Sigma-Aldrich, USA). Cells were maintained in an atmosphere of 5% CO_2_ at 37°C. Confluent monolayers of Huh-7 cells were maintained in 75 cm^2^ culture flasks (Corning, USA), trypsinized and passaged. Cell counting was carried out using Neubauer chamber (Marienfeld, Germany). Cells (1.8×10^7^) were taken and processed in two batches of 9×10^6^ cells. The cells were pelleted and washed twice with transfection medium (serum free 1X DMEM prepared in nuclease free water). The cells were finally resuspended in 0.5 ml transfection medium and 9 µg of pcDNA3HBV-1.3mer was added in one batch of cells and in another batch, the vector only control, 9 µg of pcDNA3-null was added. The two suspensions were then taken in separate Gene Pulser® cuvettes (0.4 cm electrode) (Bio-Rad, USA) and incubated at 4°C for 10 min, followed by a brief pulse at 200 V, 975 µF capacitance in Gene Pulser® II electroporator (Bio-Rad, USA). After the pulse, cells in the cuvette were re-suspended in complete medium and diluted. Cell counts were obtained using Trypan Blue (Sigma, USA) to assess the extent of cell death. The cell suspensions were plated equally in five 60 mm plates (Corning, USA) for each batch. The cells in each plate were layered with 2 ml media and incubated as described above.

Media was changed 6 h post-electroporation to remove dead cells. Spent media over the duration of the experiment after every 24 h was filtered using a 0.2 µ filter and stored at −80°C, for HBsAg detection by ELISA. Forty-eight hours post electroporation the plates were processed for transfection of HEV RNA as described previously [18]. HEV RNA–liposomal complexes were made by incubating 4 µg of HEV RNA with Lipofectamine LTX reagent and Plus reagent (Invitrogen, USA) 1∶2.5 and 1∶1 ratios respectively for 20 minutes. The monolayers were washed thrice with transfection medium and the RNA-liposomal complexes were layered over the cells. Two plates from both the batches were mock transfected with just the liposomes, hereafter named HBV-only (for pcDNA3ΔCMVHBVWT-1.3mer only electroporated cells) and pcDNA3-only (for pcDNA3-null vector only electroporated cells). The remaining plates in the two batches were named HBV+HEV (for pcDNA3ΔCMVHBVWT-1.3mer electroporated and HEV transfected cells) and HEV-only (for pcDNA3-null electroporated and HEV transfected cells). Transfection medium in all the plates was changed 4 h post-transfection with complete medium. The plates were incubated for 24 h and cells were harvested by removing the medium. The monolayer was washed with PBS followed by addition of 1 ml Trizol reagent (Invitrogen, USA) to each plate and total RNA isolated as per manufacturer’s directions. The RNA pellets were reconstituted in nuclease free water and quantified using Qubit (Invitrogen, USA) and stored in −80°C till further use.

### Replication Validation of HBV and HEV Replicons

The filtered culture supernatants collected during the experiments, as described above, were analyzed by Monolisa HBsAg Ultra kit (Bio-Rad, USA) for HBsAg detection as per manufacturer’s instructions and detected using ELISA reader (Tecan, Switzerland). HEV negative strand replicative-intermediate as well as genomic positive-strand were detected from 500 ng of the total RNA isolated from each plate by strand specific reverse transcription followed by real-time PCR [1]. Strand-specific reverse-transcription was achieved by using anti-sense primer 5187–5168 RP (5′ A A A A A C A T G A G G A A C A G C A G 3′) for genomic strand detection and sense primer 5007–5026 FP (5′G A T T G G C A T G C T A C A G G C T G 3′) for negative strand detection in combination with M-MLV reverse transcriptase (Invitrogen, USA). Primer pair 5007–5026 FP and 5187–5168 RP was used for real-time PCR amplification of 5 µl cDNA in combination with SsoFast EvaGreen master mix (Bio-Rad, USA) on CFX96 real-time machine (Bio-Rad, USA). The following temperature profile was used for the real-time PCR: Initial denaturation 98°C for 5 min; 40 cycles of 95°C for 30 s, 60°C for 30 s, 72°C for 30 s (with plate read) followed by a default melt curve. Further, replication competence of both viruses was shown by immunofluorescence assay [1]. Cells were fixed (4% paraformaldehyde for 10 min at RT), permeabilized (3 min in pre-chilled methanol at RT) and blocked with 1% BSA (Bovine serum albumin) (Sigma-Aldrich, USA) in PBST (0.05% tween-20 in Phosphate buffered saline) for 1 h followed by 5% goat serum (Abcam, USA) for 2 h. Further, cells were stained with polyclonal rabbit anti-HBsAg (AbD Serotec, USA) and monoclonal mouse anti-HEVpORF2 [19] primary antibodies followed by secondary antibody staining with goat anti-rabbit Alexa 546 and goat anti-mouse Alexa 488 (Invitrogen, USA), respectively. Cells were finally counterstained using DAPI and imaged on TE-2000 U inverted fluorescent microscope (Nikon, USA).

### mRNA Purification

mRNA was purified from total RNA using Dynabeads mRNA direct micro kit (Life technologies, USA) as per manufacturer’s directions. Before the start of the protocol, each sample was spiked with External RNA Control Consortium (ERCC) spike-in controls (Life technologies, USA) proportionate to the quantity of total RNA in each sample. Purified mRNA was quantified in Qubit and the integrity of the mRNA was checked using a Bioanalyzer 2100 (Agilent, USA) using RNA6000 pico chip and kit as per manufacturer’s instructions. The purified and characterized mRNA samples were stored in −80°C till further use.

### Library Preparation for RNA-Seq

Library preparation for RNA-Seq involved four steps including fragmentation, adaptor ligation, cDNA synthesis, and cDNA amplification. All the steps were achieved using Ion-PGM RNA-Seq kit v2 (Life technologies, USA) as per manufacturer’s instructions. Equal quantity of mRNA (150 vng) was used as input from all the samples. The amplified cDNA was quantified in Qubit, and its profile checked for size distribution and peak concentration on Bioanalyzer 2100. The prepared libraries were stored in −20°C till further use.

### Template Preparation and RNA-Seq

Amplified cDNA fragment libraries were further clonally amplified by emulsion PCR on Ion Sphere Particles (ISP’s) using Ion One touch 200-template kit v2 DL (Life technologies, USA) in Ion One Touch v1 (Life technologies, USA) as per manufacturer’s instructions. Post PCR the template positive ISP’s were recovered and were enriched to remove non-template ISP’s on Ion One Touch ES (Life technologies, USA). The enriched ISP’s were recovered and processed for sequencing using Ion PGM 200 sequencing kit as per manufacturer’s instructions. Finally the ISP’s were loaded onto a 318 chip and sequenced on Ion torrent-PGM (Life technologies, USA) using default parameters (single-end, forward sequencing). The sequences were uploaded in NCBI-Short Read Archive (SRA) under the Bioproject PRJNA222881.

### Data Analysis of RNA-Seq

Post sequencing base calling and adaptor trimming was performed on Torrent Suite version 3.6 (Life technologies, USA). The output reads were aligned and mapped using Partek Flow software (flow_base v2.2 and flow_rna_seq v1.0) (Partek, USA). The raw reads were first subjected to pre-alignment Quality assurance and quality control (QAQC) and ERCC controls QC (using TMAP aligner). Any base below Phred value of 17 was trimmed from either sides of the reads and reads shorter than 20 nt length were removed. The processed reads were aligned by a two-step protocol to hg19 reference genome, first by using Tophat2 (default parameters) and then Bowtie2 for the remaining unaligned reads, with preference set to “very-sensitive local”. The aligned reads from both the aligners were combined and the mapping quality and coverage checked by post-alignment QAQC. Aligned reads were mapped to RefSeq (2013-01-04) transcripts (RNA-Seq analysis: Partek E/M).

The mapped reads were analyzed for differential gene expression in Partek Genomic Suite v6.6 (PGS, Partek, USA). Genes were filtered into three categories based on the number of reads mapped to a gene, as low (1 to 5 reads), medium (5 to 10 reads) and high (>10 reads) expression. Gene reads were normalized by reads mapped per kilo base length of the transcript per million reads (RPKM) method and log transformed. Log transformed RPKM values of the genes were used to obtain the differential expression by Anova. The Anova lists of each comparison, HEV vs. pcDNA3, HBV vs. pcDNA3 and HBV+HEV vs. pcDNA3 were used to obtain genes with more than +/−2 fold change in expression and p value <0.05. Further the gene lists were functionally enriched using Fischer’s exact test and restricting analysis to groups with more than 2 genes. The resulting enriched function lists were filtered based on p-value <0.05. Further gene functions were analyzed using the Gene Ontology browser (Partek Genomic Suite, Partek, USA). Individual gene function and significance of the altered expression was analyzed using KEGG pathway and NCBI database.

### Real-time PCR Validation for Differential Gene Expression

Four micrograms of total RNA from each transfection i.e pcDNA3-only, HBV-only, HEV-only, HBV+HEV was used for reverse transcription using 2.5 µM oligo-dT(18) (Thermoscientific, USA) in a 25 µl reaction containing 200 U superscript III enzyme (Invitrogen, USA), DTT 5 mM (Invitrogen, USA), MgCl_2_ 1 mM (ABI, USA), dNTP 200 µM (ABI, USA) 1X RT buffer (Invitrogen, USA) and RNaseOUT 1 U (Invitrogen, USA). The reverse transcription was carried out in ABI 2720 Thermocycler (ABI, USA) with heat lid for 50 min at 42°C, and followed by inactivation at 85°C for 5 min. 5 µl of cDNA product was used for amplification in a 20 µl reaction containing 300 nM each of forward and reverse primers (Table 1), and 1X concentration of SsoFast EvaGreen master mix (Bio-Rad, USA). The reaction was carried out on a CFX96 real-time PCR machine (Bio-Rad, USA) with the following cycling conditions: Initial denaturation 98°C for 2 min; 40 cycles of 95°C for 30 s, 55°C for 30 s (with plate read) followed by a default melt curve. All genes were studied in triplicates and post read analysis for relative gene expression was carried out on Bio-Rad CFX manager software. Glyceraldehyde-3-phosphate dehydrogenase (GAPDH) was taken as a reference gene (house keeping control) and pcDNA3 as control. The normalized fold change values for each gene was represented in a bar chart.

**Table 1:** List of primers used in gene expression validation using real time PCR.

Gene Name	Forward Primer Sequence (5′-3′) [FP]	Reverse Primer Sequence (5′-3′) [RP]
MVP	GTTTGATGTCACAGGGCAAGTTCG	CTTTAGATGGAGGGCAGTGTTGG
IRF7	TGGTCCTGGTGAAGCTGG	GATGTCGTCATAGAGGCTGTTGG
TLR7	CAGGAGATTCAAGTGAAGTTGGC	GCCCATACTTGTAGCAGCTTTCA
CHUK	GATGGAATCTCTGGAACAGCGTG	CACGGTCCTGACTCTGCACAG
CARD9	GCACACGCTGAAGCTCAG	GCCAGTCCTCCTCCAGTAC
IFIH1	CCGCTATCTCATCTCGTGCTTCAG	CAGTTCAACTGCCTGCATGTTC
PDCL3	CAGCGCATCCTCCAGCAGTCA	CAGCCAGTCTCCGCCGTCTGTACA
ADCY5	GTAGGAGGATGCATGGACACACTG	GATGAGATTGCTTCCCTTGGTTC
SARM1	GGAGGCCACCATTGAGAAGATC	GGACGATGGAAATGGAAGTCA
HK3	GGAAGAGCTGGCAGTGTCTGTG	AGGCAACAGCGGTGACCA
PCYT1B	AAGAGCCGCACCATGCAGGA	CCTCGCCCAACTCTTCAGCTG
OASL	GGACCTGAGGATGGAGCAGAGAGT	GGCTCTGTAGGCAGGCACAATG
TGFB3	GAACCACCACTGCCTGACTG	CCGTGATTCTCAGAGCCAG
LCAT	GCTACCGCAAGACAGAGGACT	ACGCGGATCTGGACACCA
MGAT3	CATCCGCCACAAGGTGCTCT	CGACTTGCGCATGTGGAAG
PYDC2	AGCAGCTCAGCCAGGATG	CTCTCCCAGACAGATGCG
PRKCG	CCACTGCACCGACTTCATC	ATGCAGGCGGAACTTGTG
SUMO4	GAGACTCCGGTGTTCACCATG	CCGTGGTTCACAATAGGCTTTC
XAF1	CACCAACGACATCCAAGC	CTCCCTGGTTAGCTGTATTCCT
DENND2C	CGCCATAAACGCTTAGCACAAC	CCTTGCCAGGGAATTGTTG

## Results and Discussion

Earlier studies from serum of patients co-infected with HEV and HBV either described an increase in HBV genome copy numbers [14] or no change in copy numbers over the course of the study [15]. In our clinical investigation no significant variation in HBV copy number was observed in co-infection as compared to individuals infected with HBV alone (Figure S2A) similar to the findings of Marion-Audibert *et al* (2010) [15]. However, the copy numbers of HEV in patients with dual infection was significantly higher as compared to individuals infected with HEV only (p = 0.007) (Figure S2B). [These were analyzed on patients with HBV and HEV dual infection (n = 26, Table S1) compared to patients with either HBV only (n = 19, Table S2) or HEV only infection (n = 16, Table S3). The serum viral load analysis for these retrospective material and dual immunofluorescence staining of retrospective formalin fixed paraffin embedded liver biopsies (Table S4 and Figure S3) were carried out on material stored at the Department of Pathology, All India Institute of Medical Sciences. The necessary consent and clearance for this study (Ref. No. IEC/NP-206/2010) was obtained from Institutional Ethical Clearance Committee for research on human subjects. The patients were informed and their consent received for the nature of tests that would be done on their samples. The blood samples were collected by standard venipuncture and vaccutainer using sterile methodology, while biopsies were carried out by standard menghini’s needle/trans jugular method after consent. This information was used for their diagnosis and treatment purpose]. In dual transfection cell culture system that lacks extraneous factors (inflammatory mediators, cells of host origin, etc), no variation was observed in the copy number of either virus (Figure S2C and S2D).

**Figure 1:**
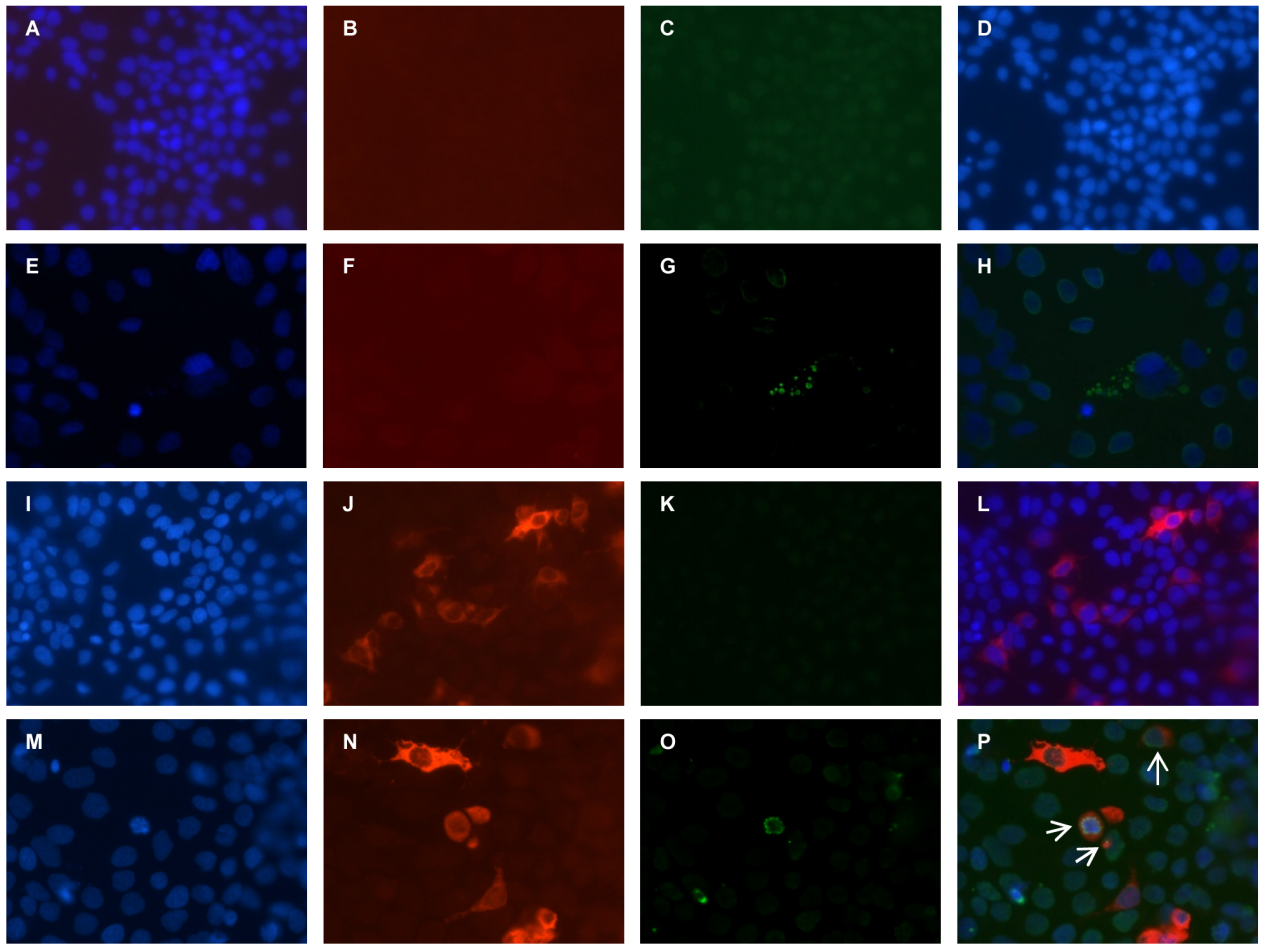
Indirect immunofluorescence for detection of HBV and HEV in dual transfected Huh-7 cell cultures. HBV (I to L), HEV (E to H) and HBV+HEV (M to P) replicon transfected Huh-7 cells were stained with anti-HBsAg rabbit polyclonal and anti-pORF2 mouse monoclonal primary antibodies, followed by Alexa 546 conjugated goat anti-rabbit and Alexa 488 conjugated goat anti-mouse secondary antibodies in an indirect immunofluorescence assay. The nuclei were counter stained with DAPI. The composite image (P) showed both HBV and HEV positive cells as well as HBV-only and HEV-only cells. Composite images H and L show positivity for HEV and HBV, respectively. Mock-transfected control showed no staining either for HBV or HEV (A to D).

This preliminary data suggested that host responses were probably involved in the rapid decompensation of the liver disease rather than the interaction between viruses per se. Further, we investigated biopsies from patients with dual infection and observed that most infected cells stain positive for both HBsAg and HEV pORF2 (Figure S3M to S3P) (Table S4). These observations led us to establish a dual replicon transfection system for assessing the changes in the host machinery in presence of both viruses. Dual and single transfected cultures were stained for HBV and HEV antigens by indirect immunofluorescence to check the establishment of infection. Most transfected cells that were positive for HBsAg also showed positive staining for HEV pORF2 in dual transfection cultures (Figure 1P). No staining was observed for HBV and HEV in mock transfection controls and HEV-only and HBV-only transfected cultures, respectively (Figure 1D, 1H and 1L).

Furthermore, HBV and HEV replication in the transfected cell cultures (HBV+HEV as well as single transfected HBV-only/HEV-only) was validated by HBsAg ELISA and negative-strand detection by strand specific real-time PCR, respectively. Supernatants from both HBV-only and HBV+HEV cultures were HBsAg positive (readout was two fold higher than water only control), while supernatants from mock transfection cultures and HEV-only cultures were negative (readout was similar to water only control). Strand specific real-time PCR for detection of HEV negative and positive strand RNA showed 0.93E+09 and 2.5E+09 copies per ml from HEV transfected and 1.07E+09 and 2.28E+09 copies per ml from HBV+HEV transfected cells, respectively.

### RNA-Seq Analysis

**Figure 2:**
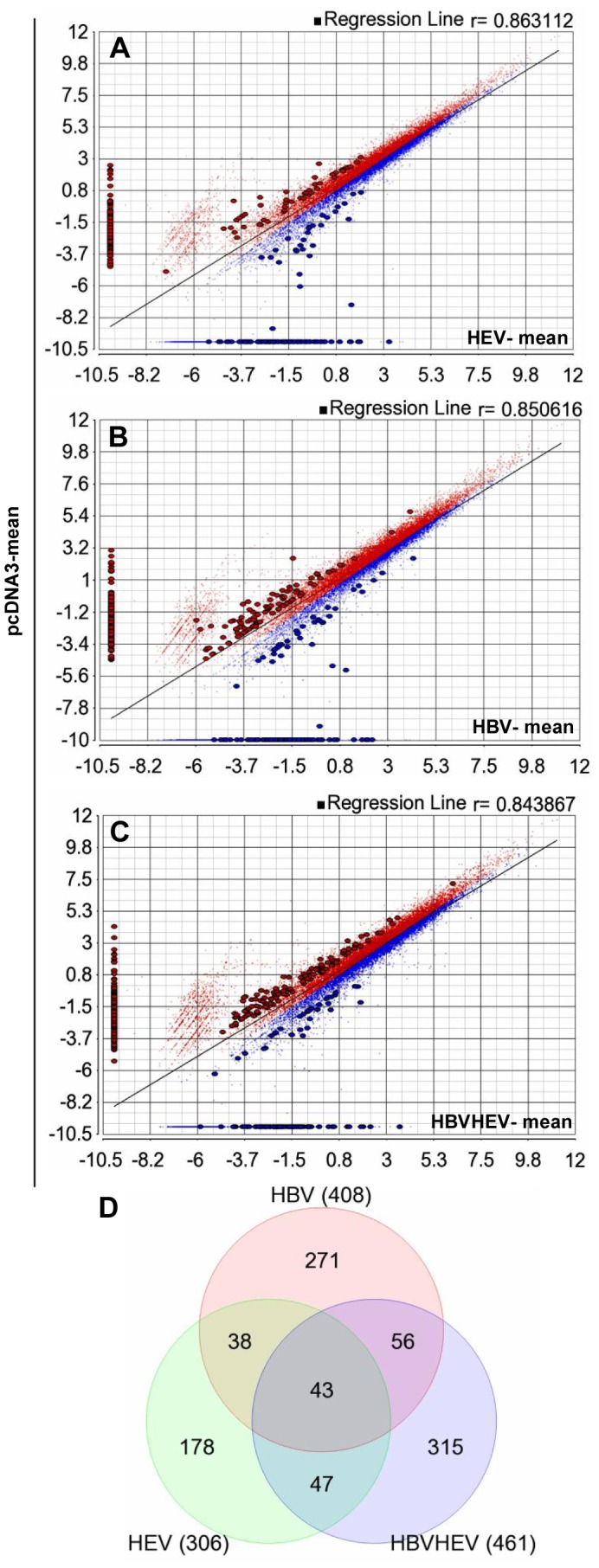
Differentially expressed genes in HEV, HBV and HEV+HBV transfected cell cultures compared to pcDNA3 vector-only control. The differential gene expression by Anova for each comparison, HEV vs. pcDNA3, HBV vs. pcDNA3 and HBV+HEV vs. pcDNA3 resulted in (A) 306, (B) 408 and (C) 461 genes with more than +/−2 fold change in expression (up regulated genes are marked in blue and down regulated genes are marked in red) and p value <0.05, respectively. (D) The Venn diagram shows common genes in the three groups.

To conduct RNA-Seq based differential expression analysis at the level of genes, highly enriched poly-A RNA was essential. Total RNA isolated from the cultures was enriched twice for poly-A RNA and >95–97% ribosomal RNA was removed. An equal amount of mRNA (150 ng) was used from all the samples during library preparation. Library concentration and size distribution was uniform across the samples, with an average mean fragment length of 193 bp (Table 2). During sequencing an average loading percentage of 79% was achieved, with 97% template positive beads (Table 2). A platform and protocol specific 24% polyclonality was observed (Table 2). Of an average 5.6 million reads, 618 million bases of sequence data was generated from each sample, with an average read length of 110 bp and average Phred quality score of 27.1 per read (Table 2). An average of 1% poor quality reads were excluded during base calling and adapter trimming, following which the reads were analyzed in Partek Flow (PF) and Partek Genomic Suite (PGS) (Partek, USA). Pre-alignment QAQC also involved analysis of the recovered ERCC spike in controls post-sequencing. An average 59 of the 92 ERCC transcripts were mapped (> = 1 count) with average Pearson correlation of 0.82 and R^2^ of 0.71 for the alignments to the actual concentrations of the transcripts (Table 3). Pre-alignment QAQC also involved trimming of low quality bases at either ends of the reads. Alignment of trimmed reads produced an overall average of 51.79% and 95.23% alignment from Tophat2 and Bowtie2, with high percentage of reads aligned uniquely to the reference genome (Table 4 and Table 5). A high mapping quality was achieved with an average mapping score of 40.2 and 20.45 from Tophat2 and Bowtie2 aligners, respectively (Table 4 and Table 5). The aligned reads were then mapped to reference transcripts and analyzed for differential expression. Averages of 15,800 genes were mapped with at least more than one read. Following differential Anova on log transformed gene RPKM values between test and control (HBV vs. pcDNA3; HEV vs. pcDNA3; and HBV+HEV vs. pcDNA3) 408, 306 and 461 genes were found to be differentially expressed by +/−2 fold with p-value <0.05, respectively (Figure 2) (Table S5). Several naturally occurring anti-sense transcripts in the gene list were not analyzed in this study given the inability of the library preparation method to discriminate sense and anti-sense transcripts.

**Table 2:** Sequencing stats and pre-alignment QAQC.

Sample name	Library fragment length	Loading (%)	Enrichment (%)	Polyclonality (%)	Test fragments (%)	Low quality (%)	Final library (reads)	Usable reads (%)	Sequence (mb)	Mean read length	Avg. read quality	%N	% GC
pcDNA3	182 bp	77	93	28	1	12	5,096,530	64	552	108	28.39	0	52.3
HEV1	189 bp	81	96	23	1	12	5,859,193	67	675	115	28.29	0	54.95
HEV2	206 bp	87	98	20	1	10	6,850,408	72	827	120	26.43	0	52.85
HBV1	236 bp	81	95	26	1	10	5,597,619	65	667	118	25.6	0	55.27
HBV2	182 bp	80	95	22	2	13	5,670,727	67	602	106	27.33	0	52.87
HBVHEV1	133 bp	75	100	23	1	15	5,331,430	64	415	77	27.39	0	54.74
HBVHEV2	223 bp	74	99	26	3	20	4,808,389	59	591	123	26.09	0	52.73

**Table 3:** ERCC spike-in mixes QAQC.

Sample name	Total number of alignments to controls	Forward strand matches	Reverse strand matches	Percent of controls present (> = 1)	Pearson correlation	Alignments to actual concentration (R2)	Correlation with sequence length	Correlation with %GC
pcDNA3	1,808	1,775	33	47.83	0.78	0.61	−9.17E−02	−1.53E−01
HEV1	6,618	6,586	32	61.96	0.86	0.74	−4.44E−02	0.08
HEV2	12,996	12,941	55	71.74	0.86	0.73	−2.07E−01	0.05
HBV1	7,873	7,849	24	60.87	0.83	0.69	0.09	0.07
HBV2	11,435	11,406	29	67.39	0.88	0.77	−8.32E−02	−1.18E−02
HBVHEV1	22,216	22,095	121	73.91	0.85	0.73	0.03	−1.23E−02
HBVHEV2	13,066	13,054	12	64.13	0.87	0.76	−1.52E−01	−1.18E−02

**Table 4:** Post-alignment QAQC using Tophat2 aligner.

Sample name	Total reads	Aligned %	Unique %	Coverage %	Avg. coverage depth	Avg. length	Avg. quality	Avg. mapping quality	% GC
pcDNA3	4,911,576	55.15	51.26	1.43	6.70 (SD 51.91)	96.93	29.6	41.41 (SD 18.47)	52.80
HEV1	5,680,301	56.01	52.64	1.72	6.87 (SD 51.67)	101.44	29.5	41.66 (SD 18.31)	54.98
HEV2	6,608,603	45.46	42.24	1.46	7.93 (SD 60.25)	106.64	27.77	41.67 (SD 18.22)	53.44
HBV1	5,392,417	41.41	38.58	1.37	5.83 (SD 71.24)	95.21	26.98	40.39 (SD 19.37)	54.95
HBV2	5,471,369	54.49	50.70	1.51	6.80 (SD 49.92)	94.25	28.57	41.41 (SD 18.48)	53.20
HBVHEV1	4,882,282	66.70	60.86	2.29	4.15 (SD 32.60)	64.29	28.52	32.59 (SD 23.58)	54.90
HBVHEV2	4,702,306	43.31	40.48	1.18	6.71 (SD 45.08)	108.31	27.56	42.15 (SD 17.81)	53.18

**Table 5:** Post-alignment QAQC using Bowtie2 of the unaligned reads from Tophat2.

Sample name	Total reads	Aligned (%)	Unique (%)	Coverage (%)	Avg. coverage depth	Avg. length	Avg. quality	Avg. mapping quality	% GC
pcDNA3	2,202,603	96.89	96.89	1.23	5.05 (SD 37.81)	126.77	27.43	19.60 (SD 14.41)	51.53
HEV1	2,498,488	96.48	96.48	1.33	5.99 (SD 182.52)	135.69	27.3	21.09 (SD 14.53)	54.73
HEV2	3,604,321	97.67	97.67	1.56	7.08 (SD 64.82)	136.02	25.73	19.33 (SD 14.38)	52.24
HBV1	3,159,660	95.31	95.31	1.30	8.08 (SD 437.10)	138.52	25.15	19.95 (SD 13.88)	55.29
HBV2	2,489,732	95.97	95.97	1.27	5.47 (SD 62.75)	123.05	26.39	20.21 (SD 14.58)	52.30
HBVHEV1	1,625,785	88.73	88.73	0.96	4.02 (SD 119.93)	104.59	26.51	22.98 (SD 14.97)	54.50
HBVHEV2	2,665,864	95.58	95.58	1.28	6.38 (SD 78.35)	135.24	25.84	19.99 (SD 14.51)	52.37

**Figure 3:**
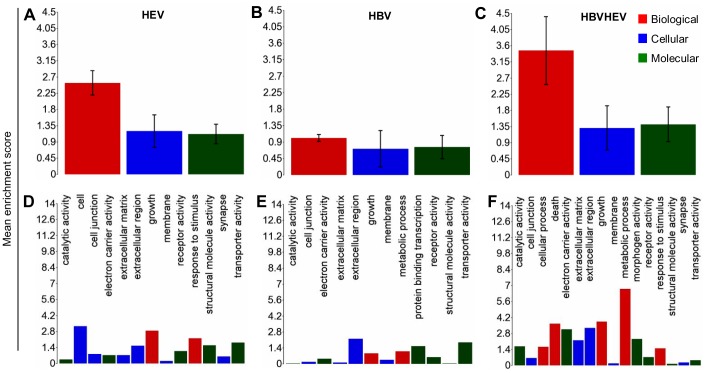
Enriched Gene ontology annotation of differentially expressed genes in HEV, HBV and HBV+HEV transfected cells. XY bar charts represent GO type (A, B and C) and functions (D, E and F) on x-axis against mean enrichment scores on y-axis. GO types include biological process, cellular component and molecular function represented in red, blue and green colors, respectively. GO enrichment of the differentially expressed genes showed a higher enrichment for HBV+HEV (C) group followed by HEV (A) and HBV (B).

**Figure 4:**
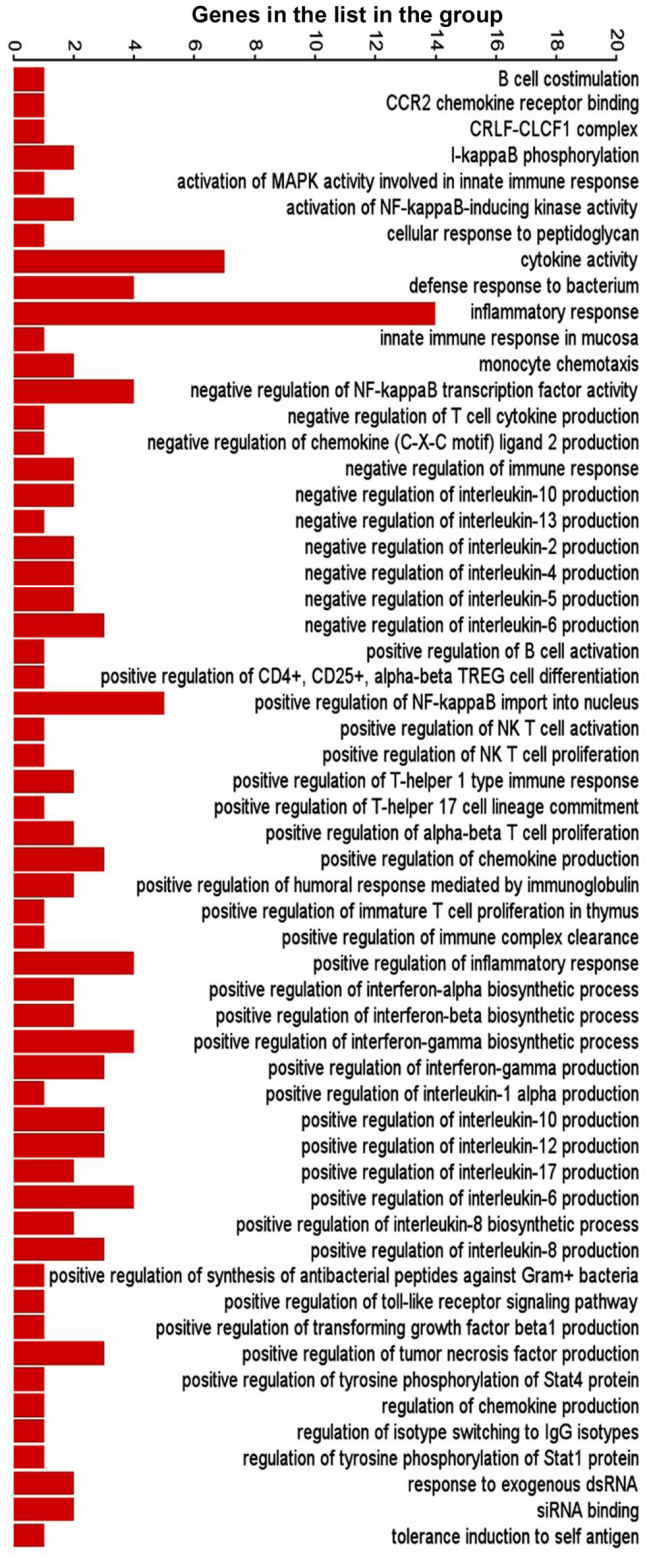
Graphical representation of host defense and survival functions derived by GO enrichment of differentially expressed genes in HBV+HEV transfected cell cultures compared to pcDNA3 control. Bars represent number of genes in the list present in each functional category.

**Figure 5:**
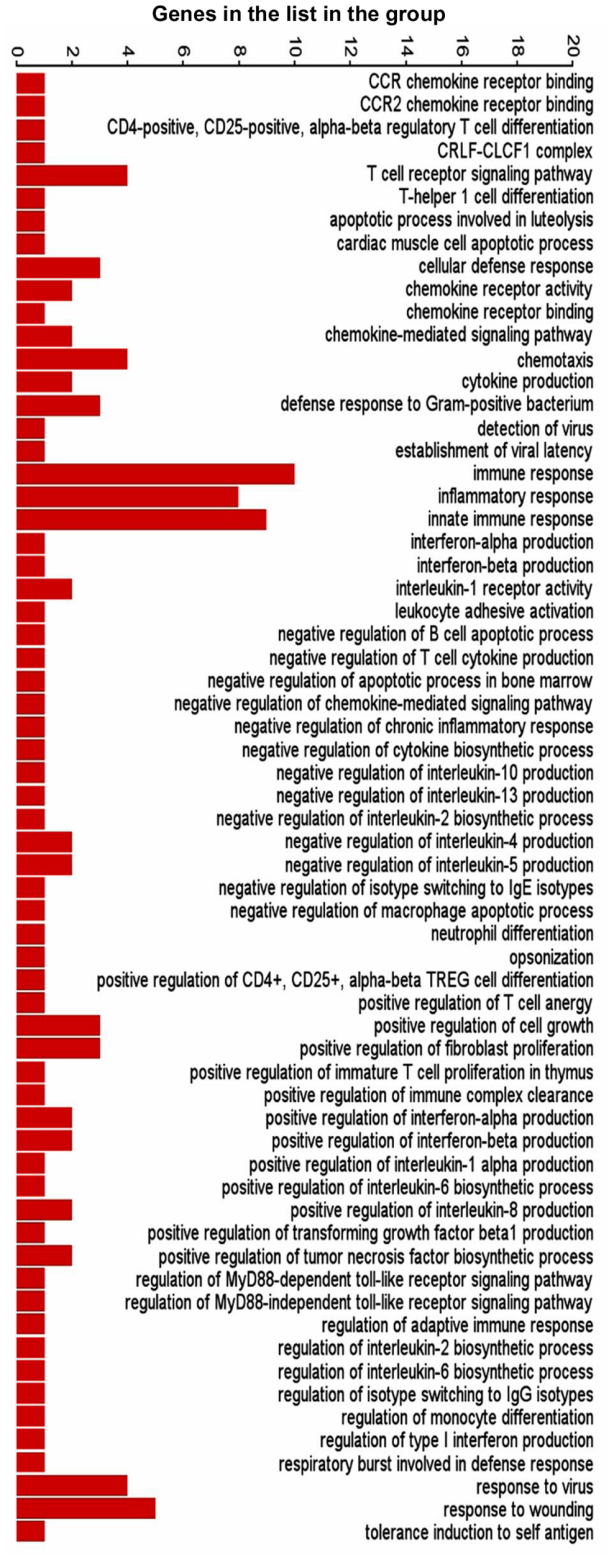
Graphical representation of host defense and survival functions derived by GO enrichment of differentially expressed genes in HEV transfected cell cultures compared to pcDNA3 control. Bars represent number of genes in the list present in each functional category.

The differentially expressed genes in the three comparison groups were enriched based on function using Gene Ontology (GO) enrichment in PGS. An enrichment p-value cut-off of <0.05 resulted in 226 out of 1244, 297 out of 1283 and 302 out of 1794 GO functions from HBV, HEV and HBV+HEV gene lists, respectively (Table S6). A significant number of genes (73 from HBV, 76 from HEV and 114 from HBV+HEV gene list), which included non-annotated transcripts, read through transcripts, long non-coding RNA and anti-sense transcripts could not be assigned any function during GO enrichment. Overall a high enrichment score with HBV+HEV>HEV>HBV was observed (Figure 3). Enrichment score for biological processes was represented high in HBV+HEV and HEV groups, while it was comparatively lower in HBV group (Figure 3). A higher enrichment for metabolic processes and cell survival was observed in HBV+HEV and HBV groups. However, in HEV a higher enrichment was observed for response to stimulus functional category (Figure 3). Three major categories based on function included, host defense and survival, signal transduction and trafficking and metabolism. A significant difference existed in the number of host defense and survival genes amongst the three groups (HBV+HEV = 57; HEV = 65 and HBV = 16 genes) (Figure 4, 5, 6 respectively) (Table S6).

**Figure 6:**
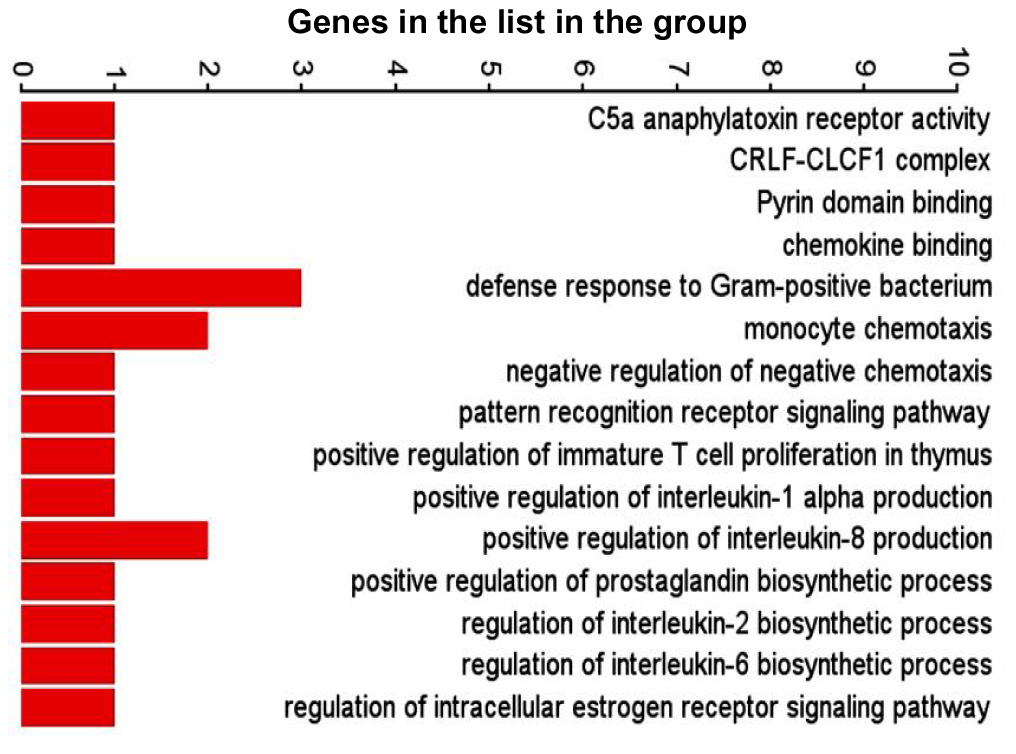
Graphical representation of host defense and survival functions derived by GO enrichment of differentially expressed genes in HBV transfected cell cultures compared to pcDNA3 control. Bars represent number of genes in the list present in each functional category.

### Validation of Differential Gene Expression by Real Time RT-PCR

**Figure 7:**
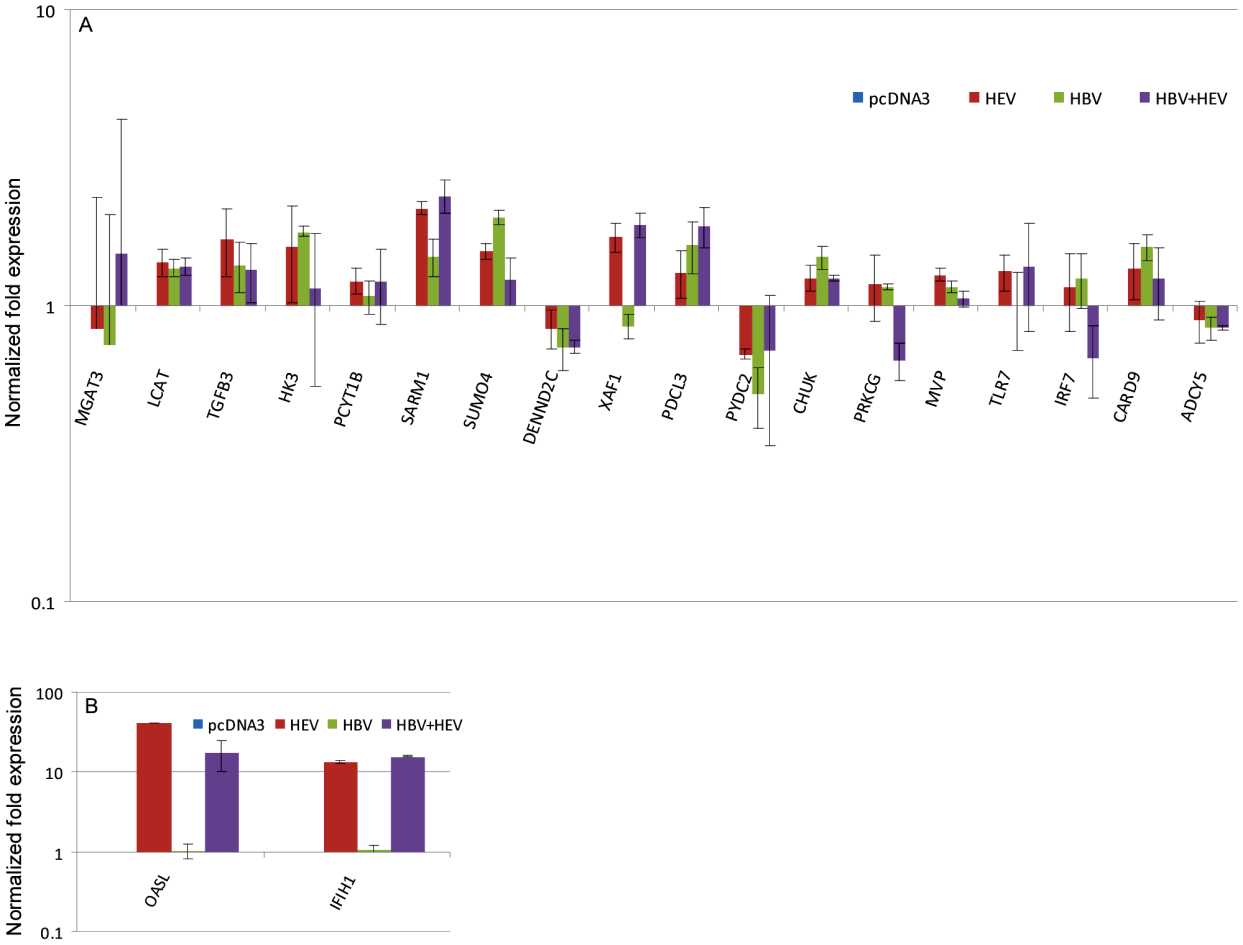
Relative quantification of differential gene expression by real-time RT PCR for validation. The XY plots represent the normalized log fold expression of genes obtained by real-time PCR as compared to pcDNA3 control (baseline represents expression in pcDNA3 control).

**Figure 8:**
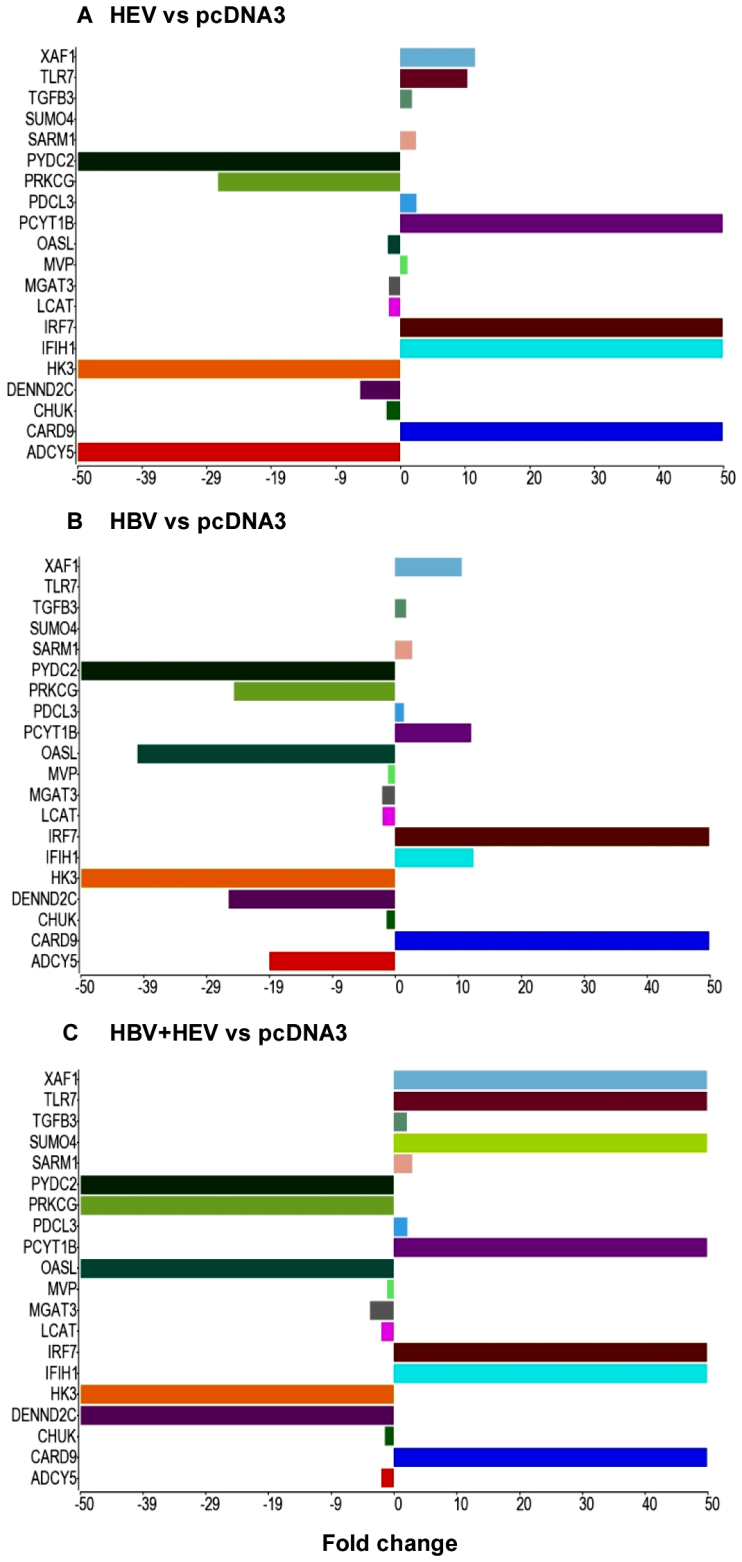
Graphical representation of RNA-Seq derived differential expression of genes that were selected for real-time PCR validation. XY bar charts represent genes selected for real-time PCR validation and their fold changes obtained in RNA-seq analysis as compared to pcDNA3 control in HEV (A), HBV (B) and HBV+HEV (C) transfected cultures. Each gene was represented by the same color in the three graphs.

Important genes based on function from the three major categories (Table 6) were validated for their differential expression using real-time PCR based relative quantification (Figure 7). A total of 20 genes were analyzed, and their real time RT-PCR gene expression profiles compared with RNA-seq data of these genes (Figure 8). Sixteen of the 20 genes had similar expression pattern as compared to RNA-seq data (CARD9, ACDY5, PYDC2, DENND2C, PRKCG, MGAT3, IFIH1, MVP, PDCL3, SARM1, SUMO4, TGFB3, TLR7, IRF7, PCYT1B and XAF1) (Figure 7 & 8). Discrepancies were seen for the remaining 4 genes (CHUK, HK3, LCAT, and OASL) that were down regulated in RNA-seq analysis but did not show differential expression or were up regulated in RT-PCR analysis (Figure 7 & 8). Majority of the genes showed similar altered expression profiles by real time RT-PCR as observed in RNA-seq analysis.

**Table 6:** Selected genes for gene expression validation by real-time PCR.

Host defense and survival		Signal transduction	Metabolism
SARM1	IRF7	SUMO4	HK3
TGFB3	CARD9	DENND2C	MGAT3
CHUK	PYDC2	PRKCG	LCAT
MVP	IFIH1	ADCY5	PCYT1B
TLR7	OASL		
PDCL3	XAF1		

### Host Defense Responses and Survival

**Figure 9:**
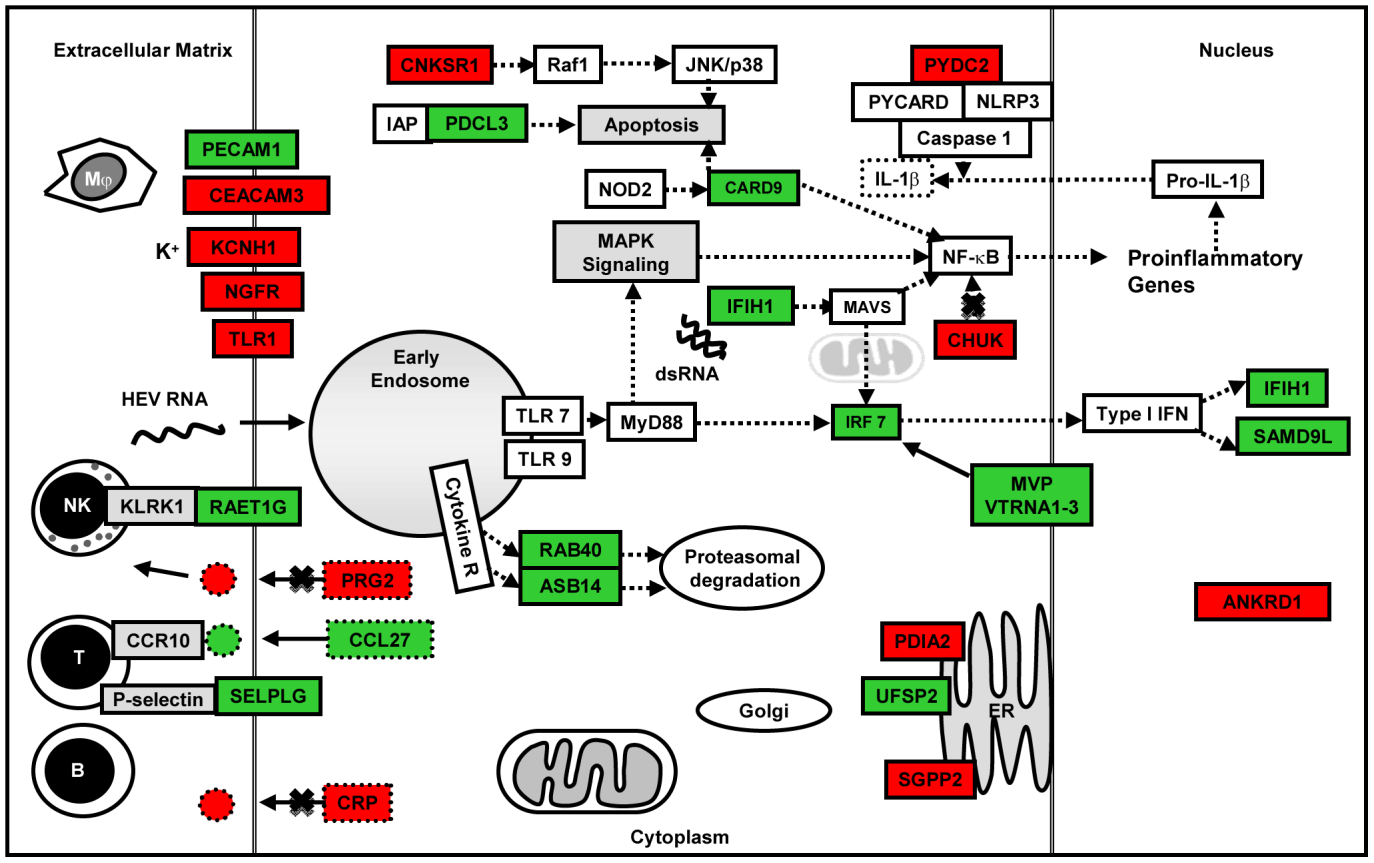
Pictorial representation of few important host defense response and apoptosis related genes that were differentially expressed (up regulated [green]; down regulated [red]) in HEV replicon transfected cell cultures compared to pcDNA3 only control.

**Figure 10:**
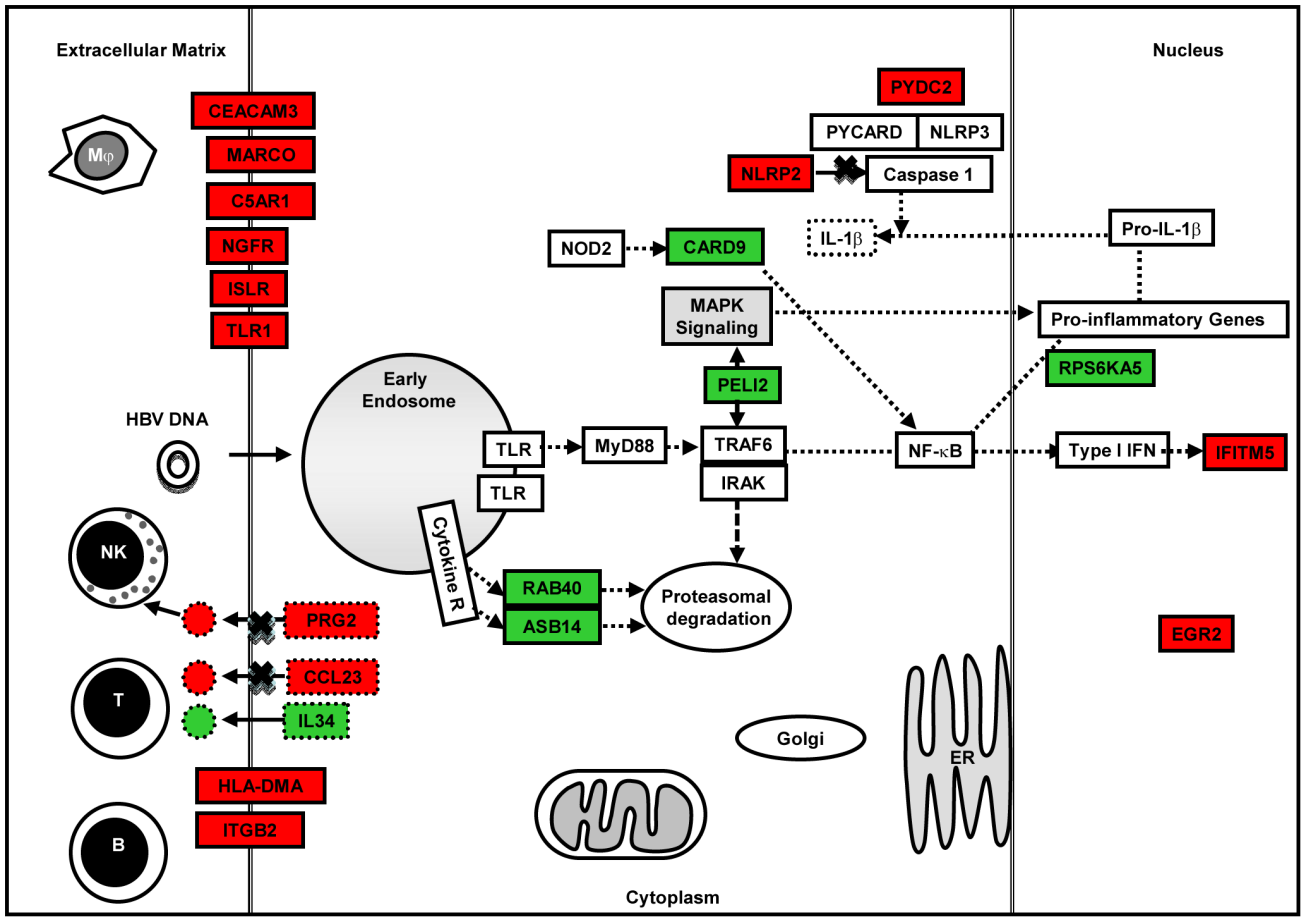
Pictorial representation of few important host defense response and apoptosis related genes that were differentially expressed (up regulated [green]; down regulated [red]) in HBV replicon transfected cell cultures compared to pcDNA3 only control.

**Figure 11:**
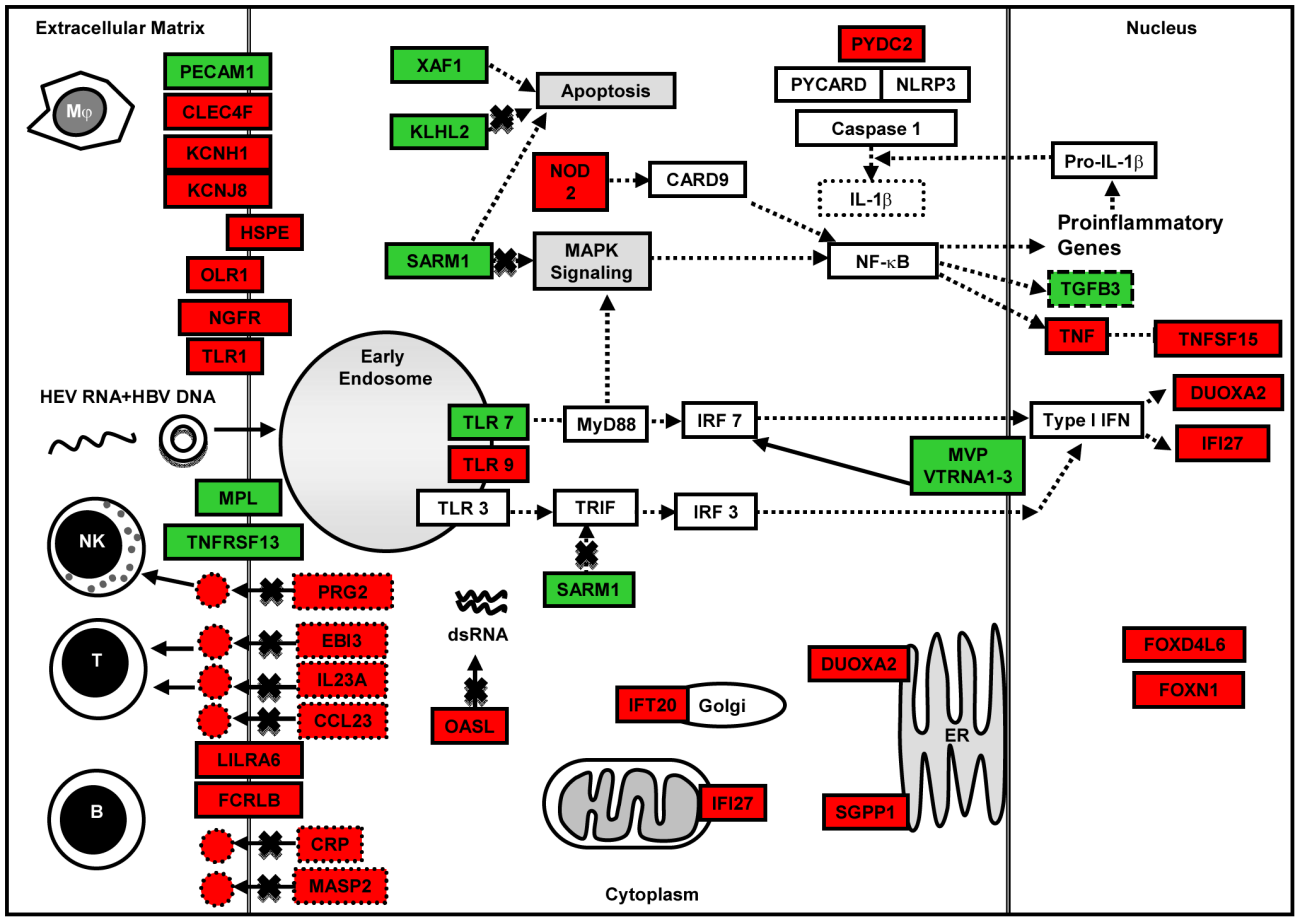
Pictorial representation of few important host defense response and apoptosis related genes that were differentially expressed (up regulated [green]; down regulated [red]) in HBV+HEV dual replicon transfected cell cultures compared to pcDNA3 only control.

Both HEV and HBV are non-cytopathic. Therefore, the first line of host innate response would determine the nature, time and type of acquired immunity that shall be triggered. In our transcriptome analysis of Huh-7 cells transfected with viral replicons, we did not use live virus, there by bypassed the innate immunity against viral proteins in the virions. Therefore, our analysis differentiates responses to only the replicon nucleic acid and stages later on. We identified differentially expressed genes that are involved in innate immune processes including pattern recognition receptor (PRR) signaling, inflammatory response, immune cell movement and interaction, antigen presentation and apoptosis (Figure 9, 10 and 11). Some of these may be towards the viral proteins that could have arisen during virus replication.

HEV-only and HBV+HEV groups showed heightened state of defense and survival responses as compared to HBV-only group (Figure 4, 5, 6, 7 & 8). HEV genome replicates through dsRNA replicative intermediates [1] that are likely to be recognized by intracellular PRRs like TLR3 or RLRs [retinoic acid-inducible gene-I (RIG-I) and melanoma differentiation associated gene 5 (MDA5). In HEV-only and HBV+HEV groups, we found IFIH1/MDA5 to be highly up-regulated as compared to HBV-only group suggesting a potential role of this gene in the activation of antiviral responses (Figure 7 & 8). Upon ligand binding, MDA5 associates with mitochondrial antiviral signaling protein (MAVS) which activates interferon regulatory factors: IRF3 and IRF7 that further activate transcription of IFNs [20]. Components of major vault protein (MVP) (a virus-induced host factor, which enhances the expression of IRF7) vault RNA 1–3 and MVP were also up regulated (Figure 7 & 8).

Gene expression of SARM1/MyD88-5 (Sterile Alpha and TIR Motif containing 1 TLR) adaptor was significantly up regulated in HEV-only and HBV+HEV groups as compared to HBV-only group (Figure 7 and 8) (Table S5). SARM1 functions as a negative regulator of TRIF-dependent TLR signaling and inhibits MAPK activation [21]. Upregulation of SARM1 has been reported in bunyavirus infection leading to neuronal death associated with oxidative stress response and mitochondrial damage [22]. Therefore, role of this adaptor in HEV super-infection of chronic HBV infection warrants further investigation. Also, endosomal TLR7 receptor and VTRNA1–3 (component of MVP) were up regulated in dual and HEV-only group that could induce IRF7 activation and type I IFN production.

NOD-like receptor pathway adaptor protein, CARD9 was up regulated in all three groups (Figure 7 and 8). CARD9 plays a critical role in NOD2-mediated regulation of NF-κB and MAP kinase signaling leading to pro-inflammatory responses (Table S5) [23], [24]. Alteration of certain genes resulting in inhibition or regulation of NF-κB was observed as in case of CHUK (conserved helix-loop-helix ubiquitous kinase)/IKK1. CHUK/IKK1, which phosphorylates NF-κB inhibitors and degrades them, thus allowing NF-κB translocation to the nucleus that leads to immune responses and protection against apoptosis was down regulated [25]. However, in real-time RT-PCR relative quantification there was upregulation of CHUK in all three groups (Figure 7). In addition, PYDC2, a negative regulator of NF-κB signaling was down-regulated in all the three groups, favoring NF-κB activation (Figure 7, 8, 9, 10 and 11) [26]. Moreover, PYDC2 is known to disrupt the interaction between PYCARD and NLRP3 that are components of multi-protein complexes called inflammosome, which is required for activation of pro-inflammatory caspases leading to maturation of cytokines like pro-IL1-β [27].

Several genes that encode either cell surface receptors or secretory factors involved in cell-cell communication and chemotaxis to the site of infection were up regulated in HEV-only group. Levels of RAETG1, a cell surface ligand for KLRK1 receptor on NK cells was up regulated that could result in NK cell-mediated cytotoxicity (Figure 9) [28]. PECAM1 (Platelet/Endothelial Cell Adhesion Molecule 1), required for leukocyte transendothelial migration [29] was up regulated in both HEV-only and HBV+HEV groups (Figure 9 and 11). Serum levels of sPECAM-1 were reported to be higher in both ALF and HCC patients [30]. CCL27, a pro-inflammatory chemotactic agent that binds to CCR10 receptor and triggers T-cell mediated inflammation [31] was up regulated (Figure 9) and so was Selectin P ligand (SELPLG) that is a high affinity counter-receptor for selectins expressed on myeloid cells and stimulated T lymphocytes and plays a critical role in leukocyte trafficking during inflammation [32].

Gene expression of several immune cell surface receptors was altered in dual infection (Figure 11) (Table S5). Myeloproliferative leukemia virus oncogene (MPL), involved in regulation of chemokine production through JAK-STAT cascade to cell proliferation [33] was up regulated. Whereas, NGFR a pro-apoptotic death receptor and OLR1 (pro-inflammatory, pro-oxidative, apoptotic scavenger receptor) were down regulated. Other genes whose expression was down regulated included SGPP1, involved in sphingolipid biosynthetic process and functions in pro-apoptotic and pro-inflammatory pathways (Table S5) [34]; Intraflagellar Transport 20 (IFT20) that forms part of a complex involved in trafficking of proteins from the Golgi body, including recycling of immune signaling components (Table S5) [35], [36]; Dual oxidase (DUOXA2), an NADPH oxidase, causes decrease in cell proliferation by G1 block, apoptosis and DNA degradation [37]. Lastly, Interferon alpha-inducible protein 27 (IFI27), plays a role in anti-viral response to positive-strand RNA viruses and activation of cysteine-type endopeptidase activity in apoptosis was down regulated (Table S5).

Transforming growth factor-β (TGF-β) is a central regulator in chronic liver disease. Liver damage induced levels of active TGF-β enhance hepatocyte destruction and mediate fibroblast and hepatic stellate cell activation [38]. In our analysis, upregulation of TGFB3 growth factor was observed in all three groups (Figure 7) (Table S5). In HBV+HEV group, TNF and TNF-inducible TNFSF15, multifunctional pro-inflammatory and pro-apoptotic cytokines were downreglated that further indicated an anti-inflammatory and pro-survival milieu. Moreover, cytokines/factors like EBI3 (component of IL-27) and IL23A (component of IL23) that could modulate T cell responses were down regulated (Table S5). Several other immune-associated genes were down regulated including cytotoxin PRG2 (NK cell activator), CLEC4F (inducible C-Type lectin involved in alpha-Galactosylceramide presentation to natural killer T (NKT) cells in the liver) and chemokine CCL23 [39], [40].

Phosducin-Like 3 (PDCL3), also known as Viral IAP-Associated Factor 1 (VIAF), functions in caspase activation during apoptosis and assists in the folding of proteins essential in regulating cell cycle progression was up regulated in all three groups (Table S5) [41]. UFM1-Specific Peptidase 2 (UFSP2), a thiol protease that specifically processes the C terminus of ubiquitin-fold modifier-1 (UFM1) to conjugate it with target proteins such as UFM1-UFBP1 that participates in preventing ER stress-induced apoptosis was up regulated in HEV-only and HBV+HEV group [42].

On the contrary, gene expression of several potential targets that might play a role in apoptosis or cell survival was down regulated in HEV-only group (ANKRD1, ATG14, CNKSR1, CDKL2, FASTKD1 and PDIA2) (Figure 9) (Table S5). Connector Enhancer of kinase suppressor of Ras 1 (CNKSR1) is essential for activation of Raf-1 that is involved in proliferation, differentiation and apoptosis. Downregulation of Raf leads to apoptosis [43]. Protein disulfide isomerase family A, member 2 (PDIA2) is a caspase substrate cleaved by caspase-3 and -7 during apoptosis. Transcriptional co-activator ANKRD1, moderately up regulates p53 activity and its downregulation could possibly prevent apoptosis.

The pattern of alteration in gene expression in HBV+HEV group was different than HEV-only group for candidate genes possibly involved in apoptosis. The up regulated genes included XIAP associated factor 1 (XAFI/BIRC4BP), which binds to and counteracts the inhibitory effect of a member of the IAP (inhibitor of apoptosis) protein family and hence promotes apoptosis. Similarly, KLHL2 (kelch-like family member) gene encodes component of Cul3-RING ubiquitin ligase complex that can bind actin. SARM1, in addition to being a negative regulator of TLR signaling is proapoptotic. Annexin A3 (ANXA3) is proapoptotic in neuronal cells but regenerative in hepatocytes. N-Acylsphingosine Amidohydrolase 2 (ASAH2) (Non-Lysosomal Ceramidase), generates ceramides during stressful stimulation and is proapoptotic. Baculoviral IAP Repeat Containing 7 (BIRC7), a member of the family of inhibitor of apoptosis proteins (IAP) and is anti-apoptotic (Figure 11) (Table S5). On the contrary, cyclin-dependent kinase 7 (CDK7) that functions as a Cdk-activating kinase (CAK) and is an important regulator of cell cycle progression was down regulated (Table S5).

A recent study investigated the role of antiviral responses in A549 cell line post HEV infection and demonstrated viral-sensing through TLR2, TLR3, and TLR4 receptors that induced MyD88 and TRIF-mediated IRF3 and NF-κB activation leading to pro-inflammatory response [44]. The limitation of this study was infection of a non-hepatocytic cell line that might not indicate the natural course of viral pathogenesis. Moreover, the cell line used is defective in TLR7 and TLR8 genes that encode receptors, which are key sensors of viral ssRNA genome in the endosomal compartment where viral capsid is uncoated [45].

### Signal Transduction and Trafficking

In either HBV or HEV infection, several signaling molecules and transcription factors were shown to be altered but the complexity and cross-talk between these potential targets is such that a specific downstream pathway that can lead to hepatitis has not been defined yet. In HEV, majority of the signaling-related studies were carried out with HEV pORF3 over-expressed *in vitro* [46], [47], [48], [49], [50] where as during virus infection the viral encoded proteins are expressed at much lower quantity. Since signaling pathways lead to pleiotropic effects, the result of an over-expressed viral protein is likely to be different than the entire complement of viral encoded proteins that can act either synergistically or antagonistically. In our transcriptome analysis, we found alteration in the expression of genes involved in signal transduction pathways including G-protein-coupled receptors (GPCR), Ras GTPases, MAPK, NF-κB, Akt and several transcription factors.

Downregulation of GPR182 receptor and downstream adenylate cyclase 5 (ADCY5) in all three groups could affect signaling by modulation of cAMP levels (Table S5). Moreover, protein tyrosine phosphatase, PTPRN2, a negative regulator of GTPase activity was up regulated that could further block cAMP pathway (Table S5). In addition, regulators of Ras GTPases, ARHGAP22 (Rho GTPase activating protein 2), DENND2C (DENN/MADD domain containing 2C, Rab guanyl-nucleotide exchange factor activity) and MPP3 (membrane-associated guanylate kinase homologs) were down regulated in all three groups (Table S5). MPP3 is a member of the MAGUK superfamily containing a conserved SH3 domain that associate with the cytoskeleton and play important role in signal transduction, regulation of cell proliferation and intracellular cell junctions.

A much robust change in expression of genes involved in signal transduction was observed in HBV+HEV group. Several GPCRs were down regulated including GPR68, RHEBL1, MCHR1 and OPN1SW (Table S5). GPR68 acutely regulates the activity of epithelial proton transport proteins. RHEBL1, GTPase activity regulates TOR signaling cascade and positively regulates NF-κB transcription activity. MCHR1 (melanin-concentrating hormone receptor 1) is an integral plasma membrane protein which binds melanin-concentrating hormone and can inhibit cAMP accumulation and stimulate intracellular calcium flux. A downstream target of cAMP is Protein kinase A (PKA), a cellular kinase that phosphorylates serine and threonine residues. PPP1R16B (protein phosphatase 1, regulatory subunit 16B) a TGF-β-inhibited protein involved in PKA-mediated moesin dephosphorylation and endothelial cell-barrier protection was down regulated (Table S5) [51]. A regulator of cAMP target, Annexin A3 (ANXA3) belongs to calcium-dependent phospholipid-binding protein family and play a role in the regulation of cellular growth was down regulated (Table S5). This protein functions in the inhibition of phopholipase A2 and cleavage of inositol 1,2-cyclic phosphate to form inositol 1-phosphate. On the contrary, a member of Ras GTPase superfamily, FGD3 (FYVE, RhoGEF and PH domain containing protein 3), involved in cell death signaling and regulation of actin cytoskeleton was up regulated (Table S5) [52].

Ras-related GTP binding protein B (RRAGB), Ras-homologous GTPases constitute a large family of signal transducers that alternate between an activated, GTP-binding state and an inactivated, GDP-binding state. Protein GRAP (GRB2-related adaptor protein), which couples signals to the Ras signaling pathway was down regulated. Protein kinase C (PRKCG) a family of serine- and threonine-specific protein kinases that can be activated by calcium and second messenger DAG was down regulated. In addition, S100A1 (S100 calcium binding protein A1), a protein involved in the regulation of cell cycle progression and differentiation was also down regulated (Table S5). Gene expression of molecules involved in G-protein coupled signaling of both arms including cAMP and phosphatidylinositol pathways were down regulated (Table S5).

TRIB1 (tribble homolog 1) that selectively controls both the extent and specificity of MAPK kinase activation of MAPK was down regulated (Table S5) [53]. In addition to being a phosphoprotein regulated by mitogenic pathways, TRIB1 appears to be key in determining cell fate in response to environmental stress. Expression of another gene that could influence MAPK cascade was down regulated in our study (Table S5). N-acylsphingosine amidohydrolase (non-lysosomal ceramidase) 2 (ASAH2) catalyzes the hydrolysis of the N-acyl linkage of ceramide to produce sphingosine. Sphingosine is a second messenger that exerts cell growth arrest and apoptosis-inducing activities where as Sphingosine-1-phosphate (S1P), a sphingolipid metabolite functions as an intra- and intercellular second messenger, promotes cell proliferation and survival.

We found upregulation of Small ubiquitin-like modifier 4 (SUMO4) that are attached to proteins and control their subcellular localization, stability, or activity. SUMO4 specifically modifies IKBA, (a member of the NF-κB inhibitor family) and negatively regulates NF-κB transcriptional activity and acts as a feedback regulator to prevent excessive activation of NF-κB [54].

### Metabolism

Major representation in metabolic processes was from the genes involved in lipid, xenobiotic and glucose metabolism. Minor groups included proteoglycan, amino acid metabolism and mitochondrial pathways. In glucose metabolism, HK3 (hexokinase 3) was uniformly down regulated across the three groups along with 2 solute carriers SLC2A2 (GLUT2) in HBV and SLC2A12 (GLUT8, 12) in HBV+HEV (Table S5). However, real-time RT-PCR validation showed upregulation of HK3 and LCAT in all three groups (Figure 7). The discrepancy especially in metabolism related genes could be due to culture-to-culture variations. In HEV-only group, ESRRG (estrogen related receptor γ) was up regulated (Table S5). The altered expression observed in the genes involved in glucose metabolism could lead to hyperglycemia and glucose intolerance in all the three groups. Facilitative glucose transporters GLUT2 and GLUT8, which are down regulated in HBV-only and HBV+HEV, respectively, can reduce the capacity of glucose uptake from blood. Upregulation of ESRRG (estrogen related receptor gamma) in HEV-only can stimulate gluconeogenesis thus contributing to hyperglycemia [55]. In contrast, several studies with long-term follow up have found no increased risk/incidence of IGT/DM in these patients [56], [57].

Several genes involved in various aspects of lipid metabolism showed altered expression across the groups. Phosphate cytidylyltransferase 1, choline, beta (PCYT1B), an enzyme required for the synthesis of Phosphatidylcholine was significantly up regulated in HEV-only and HBV+HEV group (Table S5). Also PhospholipaseA2 which catalyses the hydrolysis of phosphatidylcholine to lysophosphatidylcholine was down regulated in HBV-only group (Table S5). Earlier clinical studies documenting the spectrum of phospholipids in hepatitis B were similar to our results i.e., increased phophatidylcholine and progressively depressed levels of lysophosphatidylcholine [58], [59], [60]. As many animal studies and few human studies suggest that an increased amount of phosphatidylcholine is beneficial to the host cell in diverse disease states including viral hepatitis, this may be viewed as a beneficial host response to the viral infection. However phosphatidylcholine, which is a major component of plasma membranes, is also essential for viral replication and budding.

Downregulation of two genes ASAH2 (acylsphingosine amidohydrolase/non lysosomal ceramidase) and SGMS2 (sphingomyelin synthase2) can lead to an increase in proapototic ceramide (Table S5) [61]. Low expression of SGMS2 has also been observed to reduce fatty acid uptake and steatosis in mouse liver [62]. Acyl-CoA Synthetase Medium-Chain Family Member 1 (ACSM1, down regulated) is a fatty acid CoA ligase catalyzing the activation of medium chain fatty acids for mitochondrial beta-oxidation (Table S5). Its down regulation might contribute to steatosis, however as two other members of this family i.e., ACSM2 and ACSM3 show higher expression levels in hepatocytes [63], the significance of this alteration is uncertain. Another Acyl CoA synthetase, ACSBG1 (bubblegum family member 1, also known as lipidosin) was found to be down regulated in HEV-only group (Table S5). It mediates activation of long chain fatty acids for both synthesis of cellular lipids and degradation via beta-oxidation and plays a central role in myelinogenesis. This alteration may lead to accumulation of LCFAs and alter the cellular lipid composition.

Expression of genes involved in heparan sulphate metabolism was altered in HBV+HEV group. Upregulation of ARSG (arylsulfatase G), a lysosomal enzyme required to complete the degradation of heparan sulphate (Table S5) and downregulation of HS3ST5 (heparan sulfate 3-O-sulfotransferase) an enzyme which catalyses the rate-limiting step in heparan sulfate biosynthesis (Table S5) suggested reduction in heparan sulphate in HBV+HEV group. Given that various studies have attributed diverse functions to this proteoglycan in the setting of many viral infections, HBV and HEV in particular, the significance of this alteration in pathogenesis of viral hepatitis *in vivo* remains to be investigated. Other genes involved in glycoprotein synthesis and protein glycosylation which can have an effect on cell-cell interaction and immune responses found to be altered were B3GALT2 (a galactosyltransferase), CASD1 (capsule structure1 domain containing 1, a candidate mammalian sialate-O-acetyltransferase); both down regulated in HEV-only transfected cells and MGAT3 (Mannosyl N-acetylglucosaminyltransferase), one of the most important enzymes involved in the regulation of the biosynthesis of glycoprotein oligosaccharides was down regulated in HBV+HEV group (Table S5).

Overall, the expression of 13 genes (4 members of UGT1 family, 4 of UGT2 family, 4 of cytochrome p-450 family and NR1I3) involved in xenobiotic metabolism was altered across the three groups, 8 of these 13 were down regulated (Table S5). As liver is the major site of xenobiotic metabolism, such a downregulation would compound the cytotoxic injury ongoing due to viral hepatitis. In HBV-only group, the gene UGT1A7 was up regulated (Table S5). Several studies have documented an association between polymorphisms in this gene causing low enzyme activity with onset of HCC at an early age in viral induced hepatitis. Other than xenobiotic metabolism these enzymes are also essential for bile acid conjugation, bilirubin and steroid metabolism. Therefore, downregulation of these enzymes might have various deleterious consequences and maybe in part responsible for hyperbilirubinemia.

## Conclusion

HEV acute infection over HBV chronic disease is associated with rapid decompensation of the liver. To understand the host responses against these viruses in a dual infection, we established a transient cell culture model in Huh-7 cell line. Although the system was not an equivalent of HEV acute infection over chronic HBV disease, it allowed us to understand the initial responses by infected liver cells. We investigated the differential gene expression pattern of Huh-7 cells when transfected with HBV-only, HEV-only and both HBV and HEV replicons together using mRNA transcriptome sequencing. Functional analysis showed majority of the genes to be involved in host defense responses and survival, followed by signaling and protein modifications and metabolism. Pro-inflammatory gene alterations were more predominant in HEV-only and HBV+HEV groups as compared to HBV-only group in both RNA-seq and real time RT-PCR data. Further screening and validation of the several novel and unknown genes from this study would provide new insights into understanding the host responses and interactions during HBV and HEV co-infections.

## Supporting Information

Figure S1 **Construction of HBV replicon.** Flowchart and diagrammatic representation of the cloning strategy for construction of HBV1.3mer. The CMV promoter in pcDNA3 was removed by digestion with BglII and NotI enzymes and subsequently, ligated with BglII and NotI released fragment from pRLnull (77–1246)WTSPGE vector. The ligated vector was digested with BglII and EcoRV and end-repaired to self-ligate the vector resulting in HBV 0.3mer and represented as pcDNA3ΔCMVWTSPGE-0.3mer. Finally SacII fragment representing 1.0mer from pRLnullΔCMVWT1.86mer was ligated to SacII linearized pcDNA3ΔCMVWTSPGE-0.3mer to form the pcDNA3ΔCMVHBVWT-1.3 mer.
(TIF)

Figure S2 **Virus copy number analysis in patients and in vitro culture.** Graphs represent the average log copy numbers of HBV genomic DNA and HEV genomic RNA [patient serum samples (Table S1, S2 and S3) in top panel and transfected Huh-7 cultures in the bottom panel]. (A) Copy numbers of HBV in dual positive serum was 14.068±3.07 per ml (Table S1) and in HBV only positive serum was 13.52±3.81 per ml (Table S2) (p = 0.6) (t-test). (B) Copy numbers of HEV in dual positive serum was 19.44±5.19 per ml (Table S1) and in HEV only positive serum was 15.85±2.95 per ml (Table S3) (p = 0.007) (t-test). (C & D) No significant change was observed in copy numbers of HBV and HEV in dual (HBV+HEV) transfected as compared to single transfected cells.
(TIF)

Figure S3 **Indirect immunofluorescence for detection of HBV and HEV in liver biopsies from patients with HEV super-infection on chronic HBV infection.** Liver biopsies from HBV+HEV (M to P), HBV (I to L) and HEV (E to H) infected patients (Table S4) were stained with anti-HBsAg rabbit polyclonal and anti-pORF2 mouse monoclonal primary antibodies, followed by Alexa 546 conjugated goat anti-rabbit and Alexa 488 conjugated goat anti-mouse secondary antibodies in an indirect immunofluorescence assay. The nuclei were counter stained with DAPI. The composite image (P) shows both HBV and HEV positive cells. Composite images H and L show positivity for HEV and HBV, respectively. Biopsy from normal liver (from patients of oesophageal cancer, resected from periphery during surgery) showed no staining either for HBV or HEV (A to D).
(TIF)

Table S1 **Chronic HBV infected patients co-infected with acute Hepatitis E.**
(XLS)

Table S2 **Chronic HBV infected patients.**
(XLS)

Table S3 **HEV infected patients.**
(XLS)

Table S4 **Details of HBV, HEV and dual infected patients whose liver biopsies were included in the study.**
(XLS)

Table S5 **Gene lists with fold change and p-values of HBV, HEV and HBV+HEV groups.**
(XLS)

Table S6 **Gene Function lists obtained from differentially expressed genes in HBV, HEV and HBV+HEV groups after GO enrichment.**
(XLS)
