# Supplementary figures and images for: RNA-Seq Based Transcriptome Analysis of Hepatitis E Virus (HEV) and Hepatitis B Virus (HBV) Replicon Transfected Huh-7 Cells
Source: PLoS One. 2014 Feb 5;9(2):e87835. doi: 10.1371/journal.pone.0087835 (PMC3914852; doi:10.1371/journal.pone.0087835)

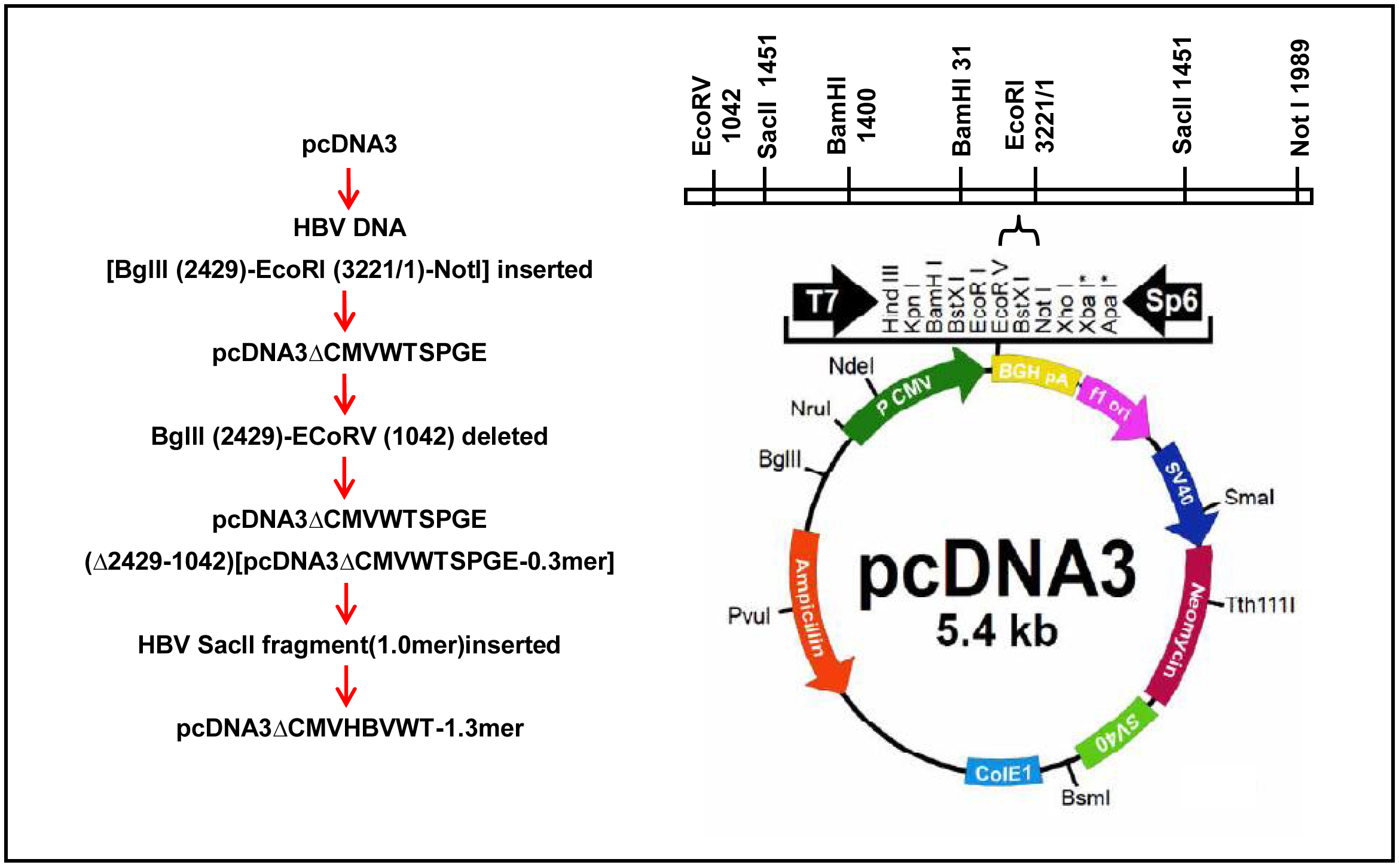

Supplement: Figure S1 — Construction of HBV replicon. Flowchart and diagrammatic representation of the cloning strategy for construction of HBV1.3mer. The CMV promoter in pcDNA3 was removed by digestion with BglII and NotI enzymes and subsequently, ligated with BglII and NotI released fragment from pRLnull (77–1246)WTSPGE vector. The ligated vector was digested with BglII and EcoRV and end-repaired to self-ligate the vector resulting in HBV 0.3mer and represented as pcDNA3ΔCMVWTSPGE-0.3mer. Finally SacII fragment representing 1.0mer from pRLnullΔCMVWT1.86mer was ligated to SacII linearized pcDNA3ΔCMVWTSPGE-0.3mer to form the pcDNA3ΔCMVHBVWT-1.3 mer. (TIF) [file pone.0087835.s001.tif]

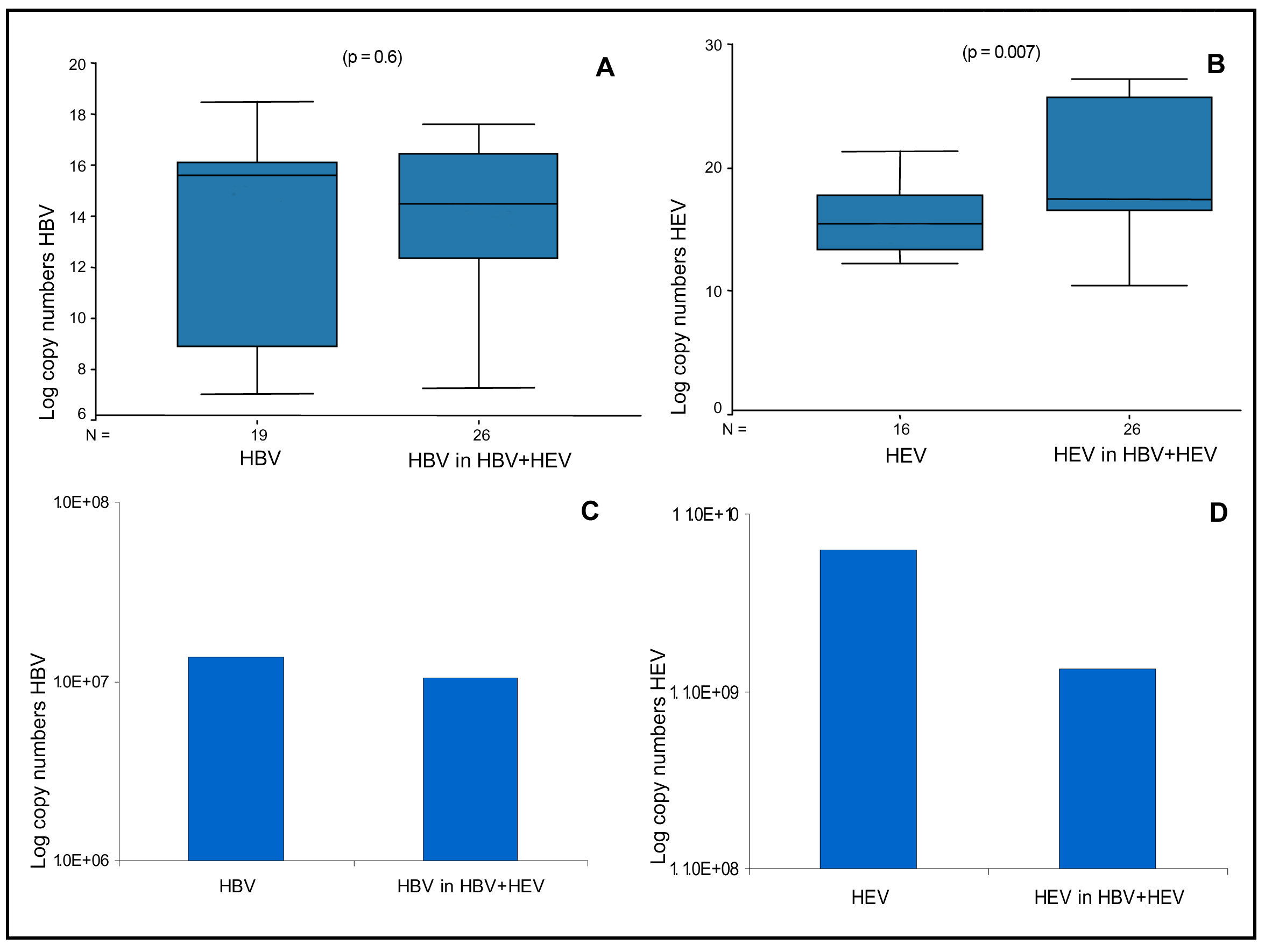

Supplement: Figure S2 — Virus copy number analysis in patients and in vitro culture. Graphs represent the average log copy numbers of HBV genomic DNA and HEV genomic RNA [patient serum samples (Table S1, S2 and S3) in top panel and transfected Huh-7 cultures in the bottom panel]. (A) Copy numbers of HBV in dual positive serum was 14.068±3.07 per ml (Table S1) and in HBV only positive serum was 13.52±3.81 per ml (Table S2) (p = 0.6) (t-test). (B) Copy numbers of HEV in dual positive serum was 19.44±5.19 per ml (Table S1) and in HEV only positive serum was 15.85±2.95 per ml (Table S3) (p = 0.007) (t-test). (C & D) No significant change was observed in copy numbers of HBV and HEV in dual (HBV+HEV) transfected as compared to single transfected cells. (TIF) [file pone.0087835.s002.tif]

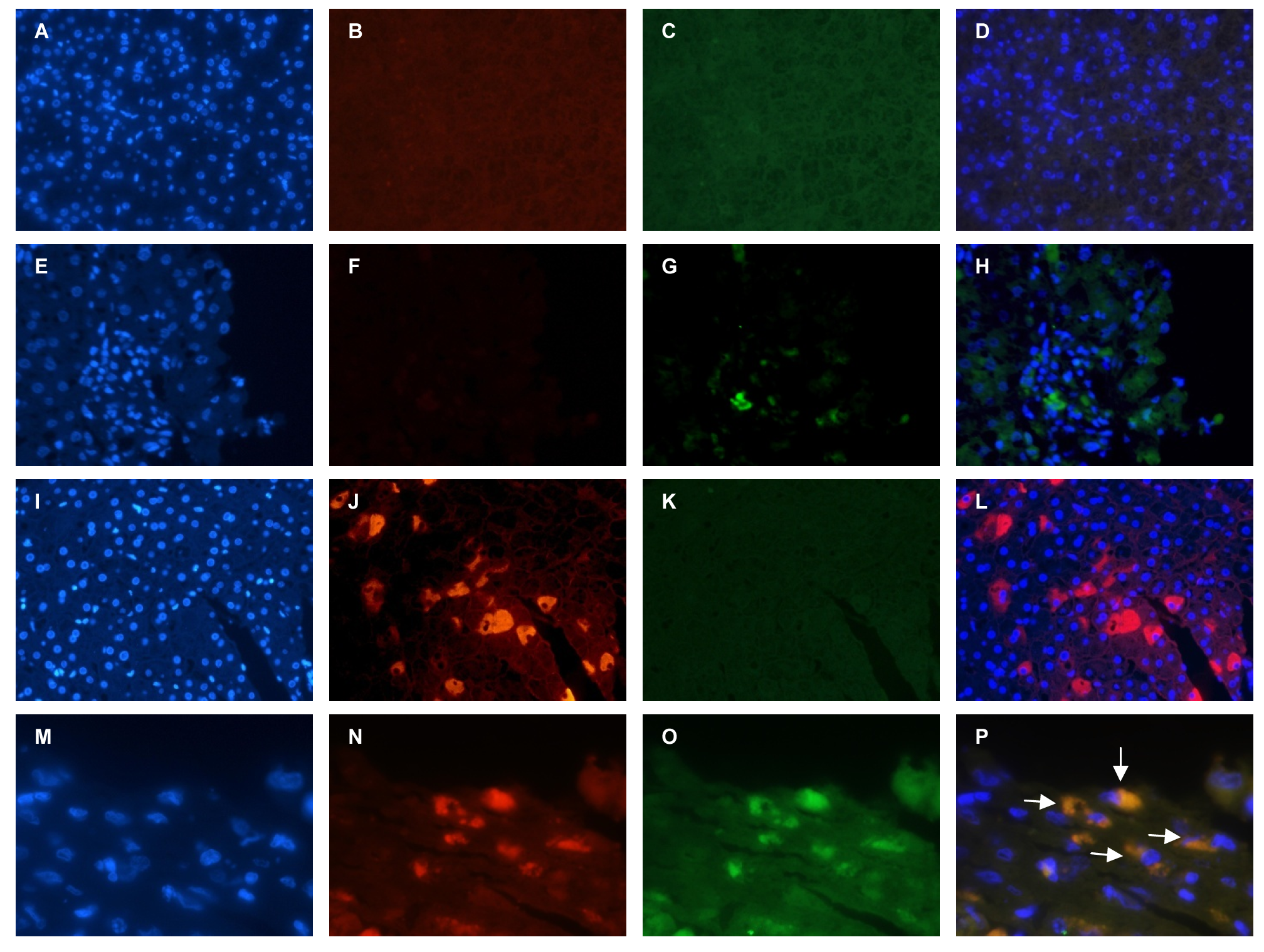

Supplement: Figure S3 — Indirect immunofluorescence for detection of HBV and HEV in liver biopsies from patients with HEV super-infection on chronic HBV infection. Liver biopsies from HBV+HEV (M to P), HBV (I to L) and HEV (E to H) infected patients (Table S4) were stained with anti-HBsAg rabbit polyclonal and anti-pORF2 mouse monoclonal primary antibodies, followed by Alexa 546 conjugated goat anti-rabbit and Alexa 488 conjugated goat anti-mouse secondary antibodies in an indirect immunofluorescence assay. The nuclei were counter stained with DAPI. The composite image (P) shows both HBV and HEV positive cells. Composite images H and L show positivity for HEV and HBV, respectively. Biopsy from normal liver (from patients of oesophageal cancer, resected from periphery during surgery) showed no staining either for HBV or HEV (A to D). (TIF) [file pone.0087835.s003.tif]
